# The IgA of hares (*Lepus* sp.) and rabbit confirms that the leporids IgA explosion is old and reveals a new case of trans-species polymorphism

**DOI:** 10.3389/fimmu.2023.1192460

**Published:** 2023-08-03

**Authors:** Ana Pinheiro, Patrícia de Sousa-Pereira, Pedro J. Esteves

**Affiliations:** ^1^ Centro de Investigação em Biodiversidade e Recursos Genéticos, Universidade do Porto (CIBIO-UP), InBIO, Laboratório Associado, Vairão, Portugal; ^2^ BIOPOLIS Program in Genomics, Biodiversity and Land Planning, CIBIO, Vairão, Portugal; ^3^ Departamento de Biologia, Faculdade de Ciências, Universidade do Porto, Porto, Portugal; ^4^ Centro de Investigação em Tecnologias de Saúde (CITS), CESPU, Gandra, Portugal

**Keywords:** leporids, hares, European rabbit, IgA, IgA hinge region

## Abstract

**Background:**

Immunoglobulin A (IgA) is the mammalian mucosal antibody, providing an important line of defense against pathogens. With 15 IgA subclasses, the European rabbit has an extremely complex IgA system, strikingly more complex than most other mammals, which have only one IgA or, in the case of hominoids, two IgA subclasses. Similar to the two hominoid primate *IGHA* genes, the expansion of the rabbit *IGHA* genes appears to have begun in an ancestral lagomorph since multiple IgA copies were found by Southern blot analysis for the genera *Sylvilagus*, *Lepus*, and *Ochotona*.

**Results:**

To gain a better insight into the extraordinary lagomorph IgA evolution, we sequenced, for the first time, expressed IgA genes for two *Lepus* species, *L. europaeus* and *L. granatensis*. These were aligned with the 15 rabbit IgA isotypes, and evolutionary analyses were conducted. The obtained phylogenetic tree shows that the *Lepus* IgA sequences cluster with and among the rabbit IgA isotypes, and the interspecies and intraspecies nucleotide genetic distances are similar. A comparison of the amino acid sequences of the *Lepus* and rabbit IgA confirms that there are two trans-species polymorphisms and that the rabbit and *Lepus* sequences share a common genetic pool. In fact, the main differences between the studied leporids IgAs reside in the characteristics of the hinge region.

**Conclusion:**

The *Lepus* IgA sequences we have obtained strongly suggest that the great expansion of the leporid *IGHA* genes occurred in a common ancestral species and was then maintained in the descendants. A strong selective pressure caused the extraordinary expansion of the *IGHA* genes but then subsided, leading to the maintenance of the acquired polymorphisms in the descendants, with little subsequent divergence. This is a unique evolutionary pattern in which an ancient gene expansion has been maintained for approximately 18 million years.

## 1 Introduction

In mammals, immunoglobulin A (IgA) is the main antibody in mucosal tissues and external secretions, providing an important line of defense against pathogens and playing a major role in the maintenance of the commensal microbiota in the intestinal tract (1). IgA is also a major antibody in the serum, being the second most abundant antibody after IgG. A considerable amount of energy goes into producing this antibody isotype since it is the most abundantly produced in the human body, seemingly indicating the importance of the IgA isotype in immune protection. Unlike other immunoglobulin isotypes, IgA can adopt multiple molecular forms, mostly monomers and dimers but also higher-order multimers linked by a J (joining) chain. In secretions, IgA is further associated with the secretory component, a polypeptide chain that confers some protection against proteolytic cleavage, a complex designated secretory IgA (S-IgA) (reviewed in (2)). IgA antibodies function by direct neutralization of pathogens or by binding to receptors, such as FcαRI, which trigger different effector functions ranging from anti- to pro-inflammatory responses (3).

IgA is present in all mammals and birds, serving as a mucosal immunoglobulin (4). Most mammals have one IgA isotype. Humans and hominoid primates (with the exception of orangutang) have two IgA isotypes, IgA1 and IgA2, which differ in the length and composition of the hinge region. The European rabbit has the most complex IgA system among mammals, with 15 IgA subclasses, which also differ mostly in the length and composition of the hinge region (5, 6). Of these, 11 IgA subclasses are expressed and show differential tissue expression (7). The two hominoid primates’ *IGHA* genes arose by gene duplication in a common hominoid primate ancestor (8). Similarly, the expansion of the rabbit *IGHA* genes appears to have begun in an ancestral lagomorph, as multiple IgA copies have been found in the genera *Sylvilagus*, *Lepus*, and *Ochotona* (5).

The order Lagomorpha encompasses rabbits, hares, and pikas. It is divided into two families, Leporidae and Ochotonidae, which diverged 30 to 55 million years ago (reviewed in (9)). The family Ochotonidae has a single genus, *Ochotona* (pikas), which includes 25 extant species. The family Leporidae is traditionally divided into two groups: rabbits, with 10 genera (*Brachylagus*, *Bunolagus*, *Caprolagus*, *Nesolagus*, *Oryctolagus*, *Pentalagus*, *Poelagus*, *Pronolagus*, *Romerolagus*, and *Sylvilagus*) and 25 species, and hares, with a single genus, *Lepus*, and 32 species (10). The *Lepus* radiation is recent, thought to have occurred within the last 4–6 million years (myr) (11), and high levels of gene flow between species have been identified (12).

The existence of multiple IgAs in lagomorph species was demonstrated by Southern blot analysis (5), but sequences of these IgAs have only been obtained for the European rabbit. To evaluate the IgA expansion in this group, we obtained, for the first time, IgA sequences from *Lepus* sp. In fact, we found seven new IgA isotypes.

## 2 Materials and methods

### 2.1 Samples, amplification, and sequencing of expressed hare IgA

Total RNA was extracted from gut tissue samples stored in RNA later at -20°C from two species of hares: two individuals of *Lepus europaeus* and one individual of *Lepus granatensis* were used in this study. These samples belong to the tissue collection of CIBIO/InBIO, Vairão, Portugal, and have been used previously for the successful amplification of other expressed genes (13). Total RNA was extracted using the RNeasy Mini Kit (Qiagen, Hilden, Germany) according to the manufacturer’s protocol, followed by first-strand complementary cDNA synthesis with the SuperScriptTM III Reverse Transcriptase Kit (Invitrogen, MA, USA) using 1µg of RNA. Primers designed in conserved regions of the CDS for all known rabbit IgA genes were used for PCR amplification of hares expressed IgA genes: Calpha_ocFw (5’ CTGCCTGATCCRGGGCTTC 3’) in the CH1 domain and Calpha_ocRv (5’ CCACGACCACAGACACGTTG 3’) in the CH3 domain (6). PCR was performed using the PCR Master Mix (Promega) with an annealing temperature of 59°C for 45 seconds and 1 minute extension, for 35 cycles. PCR products were purified (NucleoSpin Gel and PCR Clean-up kit, Macherey-Nagel, Germany) and cloned into the pGEM-T Easy vector system II (Promega, Madison, WI, USA). A total of 16 clones were selected for each hare. Sequencing was performed on an ABI PRISM 310 Genetic Analyzer (PE Applied Biosystems, MA, USA).

### 2.2 Evolutionary analysis

Of the sequences obtained in this study, those containing shift causing insertions and deletions (indels) or stop codons were eliminated from the dataset. Sequences representing putative PCR chimeras, reads composed of different parental sequences, an occurrence in multi-template PCR of similar sequences, were also eliminated. The remaining expressed IgA sequences obtained for hares (accession numbers OQ679805 - OQ679817) were aligned with Rabbit IgA1 to IgA15 sequences using ClustalW (14) as implemented in BioEdit v7.2.5 (15). Rabbit IgA1 to IgA15 sequences were obtained from GenBank (http://www.ncbi.nlm.nih.gov/genbank/) under the following accession numbers: X51647, X82108 to X82119, MH120867, and MH120868.

MEGA version 11 software (16) was used to construct a Maximum likelihood (ML) phylogenetic tree and calculate genetic distances. The phylogenetic tree was constructed using the GTR+G+I model of nucleotide substitution, which was determined to be the best-fitting model for our dataset using the Model Selection option in MEGA 11. Node support was determined from 1000 bootstrap replicate trees. This software was also used to calculate the nucleotide distances using the maximum composite likelihood method, uniform rates among sites, heterogeneous rates among lineages, and pairwise deletion of gaps options. N-linked glycosylation sites were estimated using the online tool NetNGlyc 1.0 Server, with + indicating a potential to reach the 0.5 threshold, and ++/+++ to reach the 0.75 threshold (17).

## 3 Results

**Figure 1:**
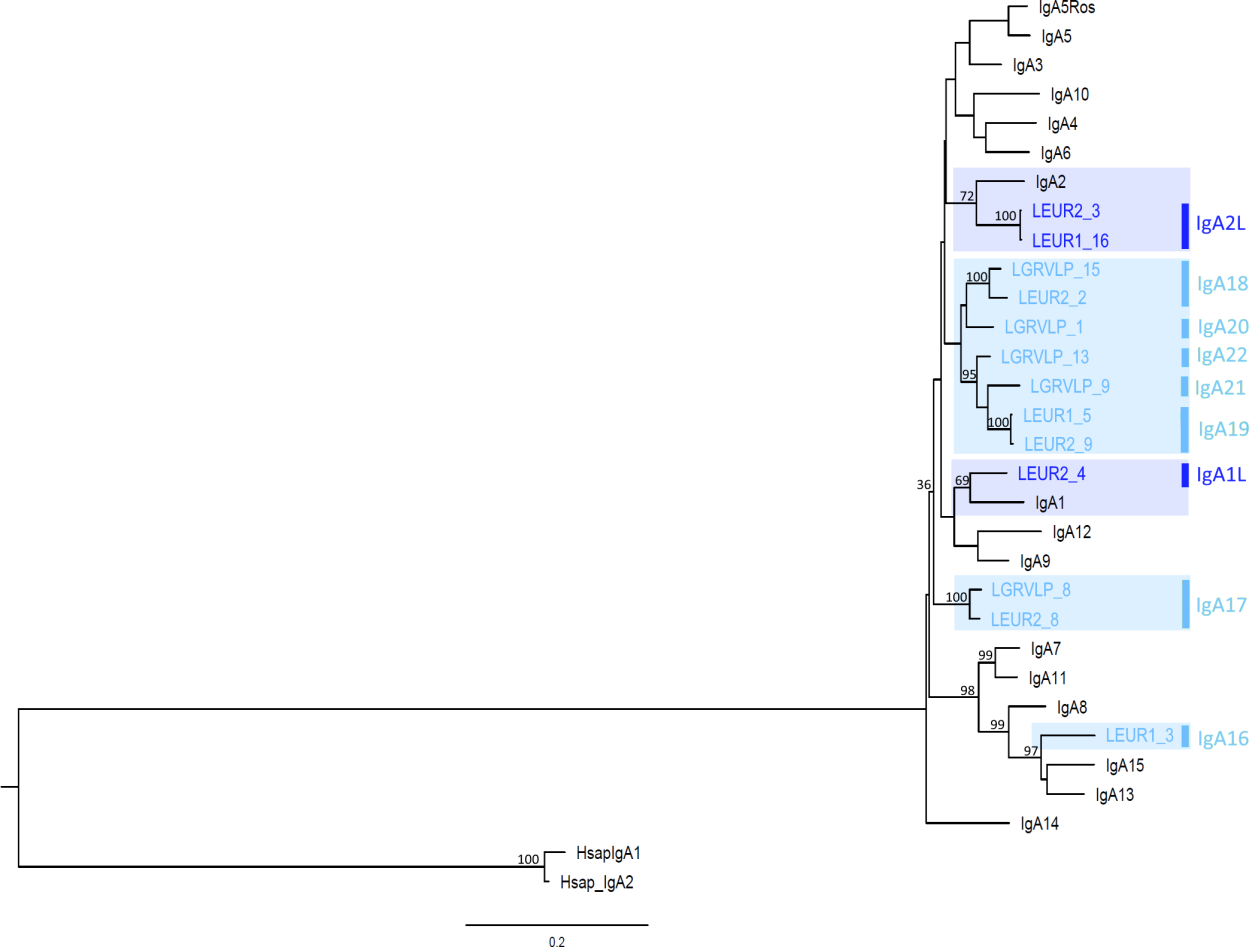
Phylogenetic tree of leporids, hares and European rabbit IgA genes. The maximum likelihood (ML) method and the GTR+G+I model of nucleotide substitution were used to obtain the IgA phylogenetic tree. Bootstrap values are indicated near the most relevant branches. The human IgA1 and IgA2 sequences were used to root the tree. Hare IgA isotypes are indicated to the right of the branch tips. Shaded in dark blue are trans-specific polymorphisms; shaded in light blue are hare-specific isotypes.

The obtained sequences were aligned with European rabbit IgA sequences, and a Maximum Likelihood (ML) phylogenetic tree was constructed. In the resulting leporid IgA ML phylogeny, some hare sequences cluster with rabbit IgA isotypes, but others are grouped within hare-specific clusters (**Figure 1**). Two of the three previously described clusters of European rabbit IgA genes (6) are shown with high bootstrap support: the IgA7 and IgA11 cluster (99 bootstrap support) along with the IgA8, IgA13, and IgA15 cluster, which includes a *L. europaeus* sequence (99 bootstrap support). The third and larger European rabbit IgA1 to IgA6, IgA9, IgA10, and IgA12 groups are also shown, but with poor bootstrap support (36 bootstrap support). This group also encompasses 12 hare sequences, and the low bootstrap probably reflects the complex relationships within it. However, subgroups with good bootstrap support were observed within this large group. Two *L. europaeus* sequences, LEGR2_3 and LEGR1_16, group with rabbit IgA2 (72 bootstrap support), and one *L. europaeus* sequence, LEGR2_4, groups with rabbit IgA1 (69 bootstrap support). Three clusters of exclusively hare sequences group *L. europaeus* and *L. granatensis* sequences: LGRVLP8 and LEGR2_8, (100 bootstrap support), LGRVLP15 and LEGR2_2 (100 bootstrap support), and LGRVLP13, LGRVLP9, LEGR1_5, and LEGR2_9 (95 bootstrap support) (**Figure 1**). It thus appears that hares share some IgA isotypes with rabbit, such as IgA2-like, IgA1-like, and IgA13/15-like, and also have hare-specific isotypes.

The genetic distances between hares *IGHA* genes (0.02–0.196; **Supplementary Table S1**) is similar to that between rabbit *IGHA* genes (0.047-0.241; **Supplementary Table S1**). The genetic distances between hares and rabbit *IGHA* genes (0.081-0.218, **Supplementary Table S1**) are in the same range as the intra-species distances. The similarity between rabbit and hares *IGHA* genes concurs to show that the IgA expansion has started in a leporid ancestor.

### 3.1 Characterization of hare IgA

**Figure 2:**
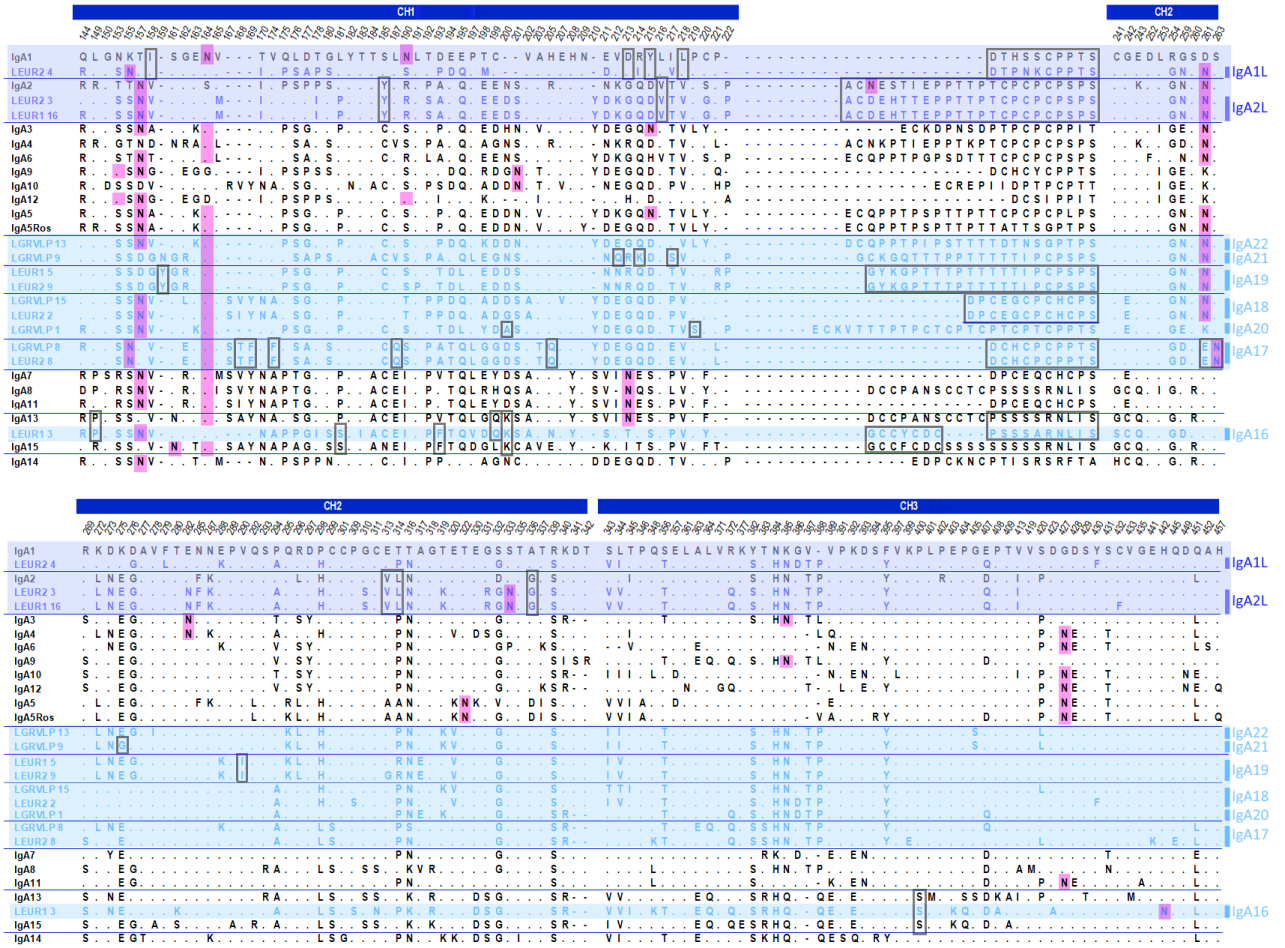
Amino acid sequence alignment of hares and European rabbit IgAs. The entire hinge region sequences are depicted. For the remaining constant region domains, only the amino acid polymorphic positions are shown. The human IgA1 numbering for these positions is shown above. Sequences are grouped according to the phylogenetic clustering. The hares IgA isotypes are indicated to the right of the alignment; trans-specific polymorphisms are shaded in dark blue and hare-specific isotypes are shaded in light blue. Isotype-specific residues and shared hinge sequences are shown in boxes. N-glycosylation sites identified using the online tool NetNGlyc 1.0 (17) are highlighted in pink. Dots (.) represent identity with the top sequence, and dashes (–) indicate gaps in the alignment.

The inferred amino acid sequences were grouped according to phylogenetic clustering and compared with rabbit IgA isotypes (**Figure 2**). The European rabbit 15 IgA isotypes differ mostly in the length and composition of the hinge region, but differences are also present in the constant domains (5, 6).

Considering the hares IgA sequences that grouped with rabbit IgA sequences, their comparison with rabbit IgA revealed similarities between the hares and rabbit sequences. The two *L. europaeus* sequences, LEGR2_3 and LEGR1_16, share a similar hinge region with rabbit IgA2, differing in only three amino acid positions (**Figure 2**). These three sequences also share unique residues in the constant domains: Y185, V216, 313VL314, and G336. Given these similarities, we consider that the LEUR2_3 and LEUR1_16 sequences represent the hares IgA2 isotype and we refer to it as IgA2L. The LEGR2_4 sequence shares with rabbit IgA1 a hinge region of similar length, that differs in three residues, and four amino acids in the CH1 domain, I158, D213, Y215, and L218 (**Figure 2**). LEGR2_4 represents the hares IgA1 isotype, hereafter referred to as IgA1L.

The LEGR1_3 sequence, which grouped with rabbit IgA13 and IgA15, shares two unique residues with both sequences along the constant domains, K200 and S400. In the CH1 domain, it also shares two residues with IgA15, S181, and F193, and two other residues with IgA13, P149, and Q199. The 17 residue-long LEGR1_3 hinge region appears to share the first seven residues of the IgA15 hinge region and the last ten residues of the IgA13 hinge region, differing from the rabbit hinges in only one amino acid for each segment (**Figure 2**). We refer to this unique hare IgA as IgA16.

As for the hare sequences that clustered in hare-specific clusters, LGRVLP8 and LEGR2_8, sequences that grouped with 99 bootstrap support share seven unique residues in the CH1 and CH2 domains: 168TF169, F174, Q187, Q205, 261EN263, and the hinge region sequence (**Figure 2**). These sequences represent a hare isotype we designated as IgA17. LGRVLP15 and LEGR2_2 (99 bootstrap support) have identical sequences and a distinctive 12-residue hinge region motif (**Figure 2**), an exclusive hare isotype that we designated as IgA18. Within the group formed by LGRVLP13, LGRVLP9, LEGR1_5, and LEGR2_9 (95 bootstrap support), LEGR1_5 and LEGR2_9 formed a subgroup (100 bootstrap support; **Figure 1**). These two sequences are identical, with unique residues Y159 and I292 and a 21-residue-long hinge region with an exclusive motif (**Figure 2**). We designated this hare isotype as IgA19. As for the LGRVLP9 and LGRVLP13 sequences, these represent the hare isotypes IgA21 and IgA22, respectively. The LGRVLP9 sequence has four unique residues, Q212, K214, S217 (CH1), and G275 (CH2), and a 22-residue-long hinge region (**Figure 2**). The LGRVLP13 sequence has a 23-residue-long hinge region similar to the IgA5Ros hinge region but differing at five amino acid positions (**Figure 2**). The LGRVLP1 sequence has two unique residues, A200 and S219, and a 26-residue-long hinge composed almost exclusively of T, P, and C (four repeats of the TCP motif), representing the hare IgA20 isotype.

Immunoglobulin heavy chains are organized into globular domains, a structure stabilized by intra-chain disulphide bonds between conserved cysteines in each domain. These cysteines, located at positions 141, 204, 266, 322, 369, and 432, are conserved in leporid IgA sequences except for the Leur1_16 sequence, which has an F432 (resulting from a single nucleotide substitution; data not shown). Glycosylation is also important for protein structure and function, and human IgA is one of the most heavily glycosylated immunoglobulins. Leporid IgAs exhibit several putative N-glycosylation sites, most of which are found in the CH1 and hinge proximal CH2, but also in the CH3 domain (**Figure 2**). One N-glycosylation site, N459, in the tailpiece, is conserved across all isotypes (data not shown) and indeed in all mammals. Notably, all leporid IgA isotypes have at least one N-glycosylation site in the N-terminal CH1 domain, most commonly N157 and/or N164. Some rabbit IgAs also have an N-glycosylation site toward the C-terminal CH1 (N213 in IgA7, IgA8, IgA11, and IgA13, or N215 in IgA3 and IgA5). Most hare isotypes, except IgA16 and IgA20, have an N-glycosylation site in the CH2 hinge proximal region (N261/263), which is also shared by four rabbit isotypes, IgA3, IgA4, IgA5, and IgA6. All hare isotypes carry an S and/or T residue in the hinge region and are therefore potentially O-glycosylated.

Sites of interaction with host receptors have been described for human IgA1. The 440PLAF443 loop, a surface-exposed CH3 domain loop, is particularly relevant for the binding of all studied IgA1 receptors, FcαRI, pIgR, and Fcα/µR (18–22). Interestingly, both hare and rabbit IgA isotypes share a conserved 440AEHF443 motif, suggesting that this loop is also important for leporid IgA function. Additionally, residues 257LL258, R382, E389, M433, and E437 are required for FcαRI binding, and residues C311, K377, and the motif 402QEPSQGTTTFAVT414 are required for pIgR interaction (18–22). In leporid IgA and concerning the FcαRI binding sites, residues 257LL258 and E437 are also conserved, while R382 has been substituted by an S382 or Y382 and M433 by a V433, and E389 is a polymorphic position. Regarding the pIgR binding sites, C311 and K377 are also present, but at position 311 an S and N residue are also observed, and K377 was substituted by E377 in IgA15. The motif between positions 402 and 414 is relatively well conserved within leporid IgA, with the exception of IgA13, IgA15, and IgA16, which exhibit more amino acid differences compared to the remaining IgAs.

## 4 Discussion

The European rabbit is the only mammal known to have 15 IgA subclasses, a much more complex system than most mammals (5, 6). Using Southern blot analysis, Burnett and co-workers (5) showed that other lagomorphs also have multiple IgA isotypes and that the duplication of the *IGHA* genes should have begun in an ancestral lagomorph at least 35 million years ago. However, to our knowledge, the sequence of these Lagomorph IgAs has never been obtained. In this study, we sequenced expressed IgA from cDNA samples of two hare species, *Lepus europaeus* and *L. granatensis*, to obtain the first sequences of hare IgA and to have a better picture of leporid IgA evolution.

Using primers designed to bind conserved regions of all rabbit IgA subclasses, we found nine IgA isotypes for the two hare species studied, six isotypes for *L. europaeus*, and five isotypes for *L. granatensis*. Using the same primer pair, we found 10 out of 15 rabbit IgAs for four European rabbits (6). A possible explanation for not finding all 15 rabbit IgA isotypes in the sampled animals was due to differences in the expression levels of the different IgAs (6). In the European rabbit, IgA expression is regulated by several elements, among which is a negative regulatory element (NRE) associated with rabbit germline *IGHA* genes. This NRE negatively regulates the Iα promoter and hs1, 2 enhancement of this promoter and may contribute to differences in the expression levels of the various IGHA genes (23). Thus, there are differences in the expression levels of the different rabbit IgA isotypes, with some of them being expressed at a much higher level while others are not expressed at all, with some inter-individual variation (6, 7). This pattern may well apply to hares, and therefore the isotypes we sequenced in this study may be the ones with higher expression levels in the individuals analyzed, while others with lower expression levels may exist and have not been detected by us. Accordingly, the isotypes we have detected are a minimum estimate of the hare IgA isotypes, and the number of *IGHA* genes will increase with the sequencing of other individuals.

The newly discovered hare sequences generally retain the key amino acids associated with IgA function and structure. Indeed, the glycosylation sites that are present in the hare IgA isotypes have a similar pattern to that observed in the rabbit isotypes. Glycosylation is very important for IgA immune function: aberrantly glycosylated IgA forms are associated with autoimmune diseases such as IgA nephropathy, IgA vasculitis, and rheumatoid arthritis (24); glycans attached to N459 can interact directly with and neutralize certain viruses (25); and finally, glycans impact the interactions of S-IgA with commensal microorganisms, thus influencing the microbiota composition and gut homeostasis (26, 27).

The presence of hare sequences that cluster with rabbit isotypes in the ML tree, the genetic distances between these hare and rabbit isotypes that are lower than those obtained between other hare or rabbit isotypes, and the sharing of characteristic amino acids between these hare and rabbit sequences suggest that the IgA1 and IgA1L, IgA2, and IgA2L isotypes are trans-species polymorphisms. A trans-species polymorphism occurs when genetic variants are maintained in the genome for a very long time and their origin predates speciation events, resulting in the sharing of alleles between related species (28). This reinforces the hypothesis that the explosion of IgA isotypes occurred in an ancestral lagomorph at least 35 million years ago (5). Some of the isotypes present in the leporid ancestor (around 18 million years ago, (29)) have been retained in descendant species such as European rabbit and hares. Indeed, trans-species polymorphisms have also been observed between rabbit and hares in other antibody genes such as *IGHV* or IGCK1 (30, 31).

We further found nine hare-specific isotypes. As expected, considering the recent *Lepus* radiation (11) and the reported high level of gene flow between hare species (12), some of these isotypes, the IgA17 and IgA18, are shared between the two studied hare species. More interestingly, the hare IgA isotypes are not very different from the rabbit-specific isotypes. The calculated interspecies genetic distances of hares IgA to rabbit IgA isotypes are in the same range as the intraspecies genetic distances. In the constant domains, apart from a few isotype-specific positions, there are no residues specific to rabbit or hare isotypes. The major differences between rabbit IgA isotypes are in the composition and length of the hinge regions (5, 6), which is also true for the leporid IgA isotypes studied. The differences in hinge characteristics confer different susceptibilities to bacterial proteases. The longer hinge region of human IgA1 is associated with susceptibility to cleavage by certain bacterial proteases, whereas the short hinge region of human IgA2 is resistant to bacterial proteases cleavage (18, 32). Studying the susceptibility of different rabbit IgA hinge regions to bacterial proteases, de Sousa-Pereira and co-workers (33) showed that not only the hinge length but also the hinge composition can render this region resistant to bacterial proteases cleavage. Thus, the leporids’ different hinge region composition may have evolved to confer resistance to a diversity of pathogens, as has been suggested before (5). It has been suggested that mammalian IgAs have evolved under pathogen pressure (34, 35). A similar pathogen pressure may have influenced the unusual duplication of leporid IgA genes. Additionally, it would be very interesting to study the IgA genes of American and African lagomorphs to test the effects of different selective pressures on shaping the IgA diversity of different species. In fact, our studies of leporid VDJ genes (36–38) show that American leporid species use a different VH repertoire than European species, which may be related to immune adaptation to different environmental conditions, such as distinct pathogenic agents. Sequencing of American and African lagomorph IgA may reveal new isotypes, adapted to pathogens from different environments. Despite the greater susceptibility to bacterial proteases, extended hinges are thought to bind with greater avidity to antigens spaced relatively far apart (39), and, hence, it is advantageous to retain these long hinge regions, as is, probably, illustrated by the fact that the majority of rabbit and hares IgA isotypes have long hinges.

The hare sequences obtained in this study allow us to propose a better-supported evolutionary history for the lagomorph *IGHA* genes. The low differentiation between the European rabbit and hare IgAs and the presence of trans-species polymorphisms strongly suggest that the great expansion of leporid *IGHA* genes occurred in an ancestral species and was then maintained in the descendants. An ancestral leporid suffered a major selective pressure that caused the extraordinary expansion of the *IGHA* genes that we now observe in modern lagomorphs. This selective pressure has then subsided, leading to the maintenance of the acquired polymorphisms in the descendants with little subsequent divergence. This is in striking contrast to the pattern observed for other related leporid immune system genes like IgG or the polymeric immunoglobulin receptor (pIgR). The leporid IgG and pIgR are overall conserved genes, but several diagnostic positions between species have been identified (40, 41), showing that these genes are evolving independently in the leporid lineages.

## Data availability statement

The datasets presented in this study can be found in online repositories. The names of the repository/repositories and accession number(s) can be found in the article/**Supplementary Material**.

## Ethics statement

These samples belong to the CIBIO/InBIO, Vairão, Portugal, tissue collection, were obtained by local hunters, and have been previously used for the successful amplification of other expressed genes (13). For this reason, this study did not require revision or approval by an ethics committee.

## Author contributions

PS-P carried out the laboratory work. AP analyzed the data and drafted the manuscript. AP and PE conceived the study. All authors contributed to the article and approved the submitted version.
